# Interference with mutagenic aflatoxin B1-induced checkpoints through antagonistic action of ochratoxin A in intestinal cancer cells: a molecular explanation on potential risk of crosstalk between carcinogens

**DOI:** 10.18632/oncotarget.8914

**Published:** 2016-04-22

**Authors:** Juil Kim, Seong-Hwan Park, Kee Hun Do, Dongwook Kim, Yuseok Moon

**Affiliations:** ^1^ Laboratory of Mucosal Exposome and Biomodulation, Department of Biomedical Sciences and Medical Research Institute, Pusan National University School of Medicine, Yangsan, South Korea; ^2^ National Institute of Animal Science, RDA, Wanju, South Korea; ^3^ Research Institute for Basic Sciences and Immunoregulatory Therapeutics Group in Brain Busan 21 Project, Pusan, South Korea

**Keywords:** aflatoxin, ochratoxin, intestinal cancer cells, p53 protein, Mdm2 protein

## Abstract

Foodborne aflatoxin B_1_ (AFB_1_) and ochratoxin A (OTA) cause genotoxic injury and subsequent tumor formation. As a biomarker of oncogenic stimulation by genotoxic mycotoxins, p53-triggered Mdm2 was assessed in intestinal cancer cells. AFB_1_ increased Mdm2 reporter expression in a dose-dependent manner. However, this was strongly antagonized by OTA treatment. As a positive transcription factor of Mdm2 expression, p53 levels were also increased by AFB_1_ alone and reduced by OTA. With marginal cell death responses, AFB_1_ induced p53-mediated S phase arrest and cell cycle-regulating target genes, which was completely suppressed by OTA. Although enterocyte-dominant CYP3A5 counteracted AFB_1_-induced DNA damage, expression of CYP3A5 was decreased by OTA or AFB_1_. Instead, OTA enhanced expression of another metabolic inactivating enzyme CYP3A4, attenuation of formation of AFB_1_-DNA adduct and p53-mediated cell cycle checking responses to the mutagens. Finally, the growth of intestinal cancer cells exposed to the mycotoxin mixture significantly exceeded the expected growth calculated from that of cells treated with each mycotoxin. Although AFB_1_-induced mutagen formation was decreased by OTA, interference with checkpoints through antagonistic action of OTA may contribute to the survival of tumor cells with deleterious mutations by genotoxic mycotoxins, potently increasing the risk of carcinogenesis.

## INTRODUCTION

Several food-contaminating mycotoxins including aflatoxins and ochratoxins have been identified by the International Agency for Research in Cancer (IARC) as harmful carcinogens that potently promote tumor development in several organs including the liver and kidney of mammals [1, 2]. Among the carcinogenic mycotoxins, aflatoxin B1 (AFB_1_) has been regarded as a representative orally ingested carcinogen in humans, and is thus classified as a Group 1 carcinogen by the IARC [3, 4]. AFB_1_ mainly produced by the fungi *Aspergillus flavus* and *Aspergillus parasiticus* is highly bioaccumulative due to the formation of DNA adducts [5], and chronic exposure to lower levels of AFB_1_ is a major risk factor for human hepatocellular carcinoma (HCC) [6]. AFB_1_ can be metabolically converted into a mutagenic reactive exposide by cytochrome p450 mono-oxygenases, and induces the transversion of G to T within codon 249 of the tumor suppressor *p53* gene [7]. Moreover, AFB_1_ exposure and hepatitis B virus infection are associated with synergistic point mutations in the human *p53* gene [8].

Ochratoxin A (OTA) produced by *Aspergillus* and *Penicillium* fungi has been classified as a possible human carcinogen (Group 2B) by the IARC [9, 10]. The major target organ of OTA toxicity in experimental animals is the kidney, and endemic nephropathies affecting livestock as well as humans have been associated with OTA exposure [11]. In addition to nephrotoxicity, acute exposure to OTA can trigger apoptosis in various organs and tissues including the liver [12], gastrointestinal tract [13], and lymphoid tissues [14], thereby accounting for the multi-organ toxicity of this mycotoxin. In terms of carcinogenesis, OTA can be genotoxic following oxidative metabolism via direct (guanine-specific covalent DNA adduction) or indirect (reactive oxygen species-induced DNA damage) mechanisms of action [15, 16].

In response to DNA damages by mutagens, p53 is the first identified and best known tumor suppressor that controls cell cycle checkpoints and mediators of apoptosis [17]. Cells with functional p53 are arrested in the G_1_ or G_2_ phase in response to DNA damage. This is thought to allow damaged DNA to be repaired before proceeding to the next phase of the cell cycle [18]. Therefore, p53 protects cells from injury-induced genome instability by suppressing the proliferation of damaged cells [19]. Moreover, activated p53 serves as a critical fail-safe factor that prevents the expansion of potential cancer clones. Thus, the abolishment of p53 function by a mutation appears to be a highly selective event in the evolution of cancer [20, 21].

As an important negative regulator of the p53 tumor suppressor, murine double minute 2 homolog (Mdm2) controls p53 through two negative feedback modes. First, Mdm2 binds to the transactivation domain of p53 and prevents it from serving as a transcriptional activator. Second, Mdm2 as an E3 ubiquitin ligase mediates the proteasome-mediated degradation of p53 protein [22]. Functionally, Mdm2 overexpression is associated with formation of tumors with a higher degree of invasiveness, more advanced stages, greater metastatic potentiality, and resistance to chemotherapeutic agents as well as radiation [23]. In particular, Mdm2 interplays with p53 to ensure that cells are able to respond rapidly and appropriately to a wide range of genotoxic stresses, and interaction between these two factors has been identified as a predictive biomarker of carcinogenesis [24, 25].

The coexistence of many mycotoxins in environmental samples has been reported worldwide [26, 27]. For instance, 38% of the samples tested in the recent worldwide analysis of grain-based materials were found to be co-contaminated with more than two mycotoxins [27]. Although the majority of samples can comply with the most stringent guidance values, the combined toxic effects of multiple mycotoxins warrant consideration in humans. In responsive to mycotoxins, the gut epithelium acts as a barrier that senses early external insults from contaminated food-derived matrices and transmits sentinel signals to cells. Subsequently, a broad range of mucosal responses such as epithelial inflammatory diseases and carcinogenesis are initiated [28, 29].

Using an enterocyte-based reporter system, our laboratory has developed *in vitro* methods designed to identify mycotoxins associated with specific biological actions or gene regulation patterns [30, 31]. Based on the assumption that genotoxic mycotoxins may lead to oncogenic stimulation via p53, the present study was conducted to assess p53-promoted oncogenic Mdm2 expression in intestinal cancer cells. In particular, the combined actions of the two mycotoxins, aflatoxin B1 and ochratoxin A, were also evaluated in terms of p53-linked cell homeostasis including cell cycles and growth regulation.

## RESULTS

### Antagonistic action of OTA against AFB_1_–induced p53 and Mdm2 expression

**Figure 1:**
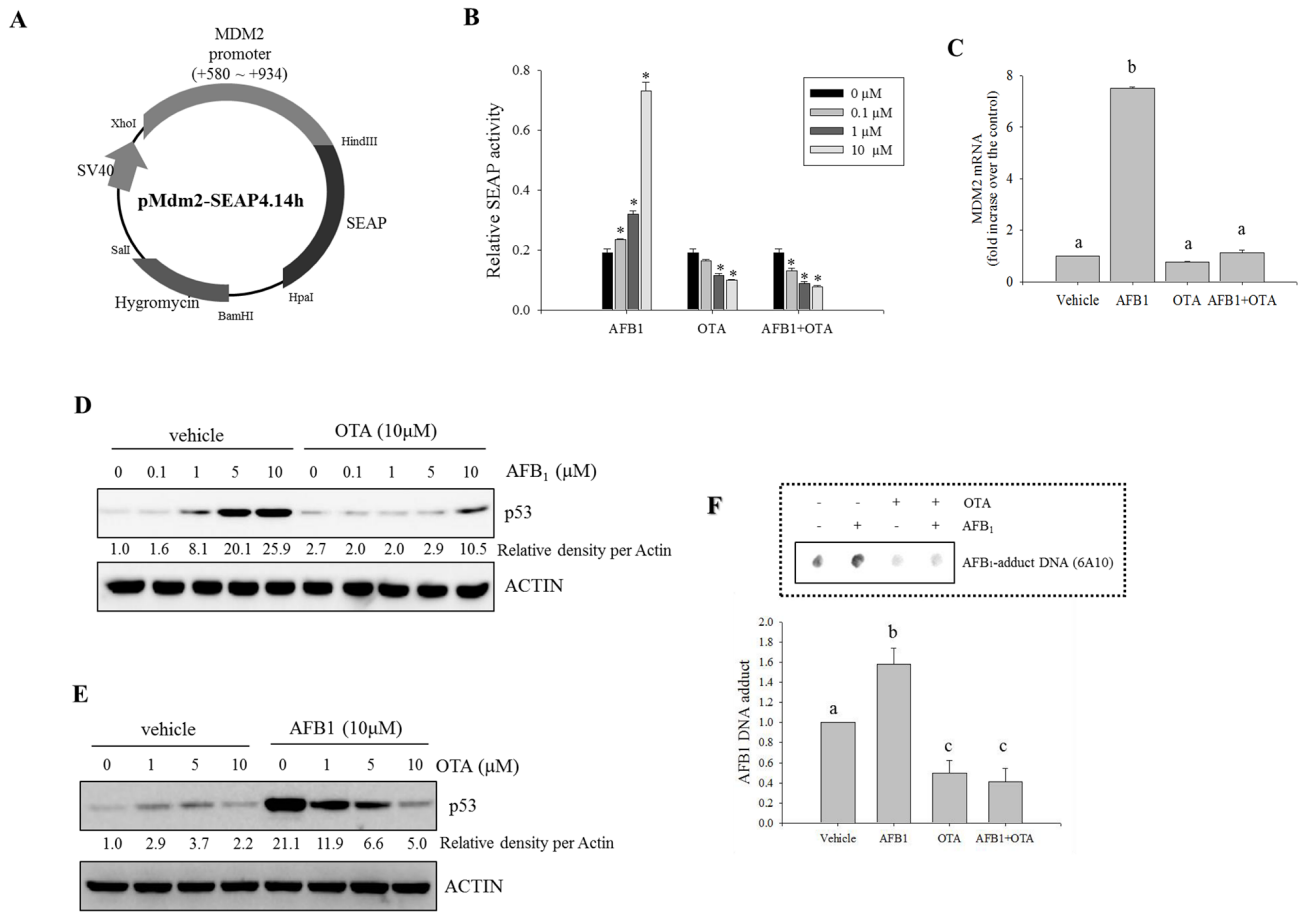
Effects of carcinogenic mycotoxins on Mdm2 and p53 expression in human intestinal cancer cells **A.** Map of the pMdm2-SEAP4.14h vector. **B.** HCT-8 intestinal cancer cells stably transfected with pMdm2-SEAP4.14h were treated with different combinations of two mycotoxin (AFB_1_ and OTA) for 24 h. SEAP activity in the culture medium was assessed. An asterisk (*) indicates a significant difference compared to each control group treated with vehicle (DMSO) alone (*p* < 0.05). **C.** HCT-8 cells were treated with AFB_1_ (10 μM), OTA (10 μM), or a combination of the two compounds for 24 h. mRNA expression of each gene was measured using real-time PCR. Different letters over the bars representing the standard deviation indicate significant differences between the two groups according to unpaired matched comparisons (*p* < 0.05). **D.** HCT-8 cells were treated with different concentrations of AFB_1_ in the presence or absence of OTA for 18 h. Total cell lysates were subjected to Western blot analysis. **E.** HCT-8 cells were treated with various concentrations of OTA in the presence or absence of AFB_1_. Total cell lysates were subjected to Western blot analysis. **F.** HCT-8 cells were treated with AFB_1_ (10 μM), OTA (10 μM), or a combination of the two compounds for 48 h. AFB_1_-DNA adducts were detected in the genomic DNA of HCT-8 cells exposed to the mycotoxins using an immunodot-blot assay. The lower graph presents the relative quantitative analysis data of the dot blot. Different letters over the bars representing the standard deviation indicate significant differences between the two groups according to unpaired matched comparisons (*p* < 0.05).

Based on the assumption that genotoxic stress induces DNA damage via p53-linked pathways, p53-promoted Mdm2 expression was monitored in human intestinal cancer cells exposed to the genotoxic mycotoxins. We constructed a proto-oncogene Mdm2 promoter-linked SEAP reporter plasmid (pMdm2-SEAP4.14h) that was used to stably transfect HCT-8 human intestinal cancer cells (Figure 1A). HCT-8 cells serve as a model for diverse mucosal diseases [32, 33]. Moreover, the proximal part of the gastrointestinal tract from which HCT-8 cells are derived is the first target organ of exposure to the majority of ingested mycotoxins [34, 35]. In response to AFB_1_ treatment, Mdm2 promoter activity was increased in a dose-dependent manner (Figure 1B). In contrast, the treatment with OTA decreased Mdm2 transcriptional activity in both the presence and absence of AFB_1_ (Figure 1B), demonstrating the negative regulatory action of OTA against Mdm2 production. Moreover, OTA treatment suppressed AFB_1_-induced Mdm2 mRNA in HCT-8 cells (Figure 1C).

As a potent upstream signaling mediator of Mdm2 expression, the production of p53 was also assessed in presence of the oncogenic mycotoxins. Treatment with AFB_1_ alone increased p53 protein expression in a dose-dependent manner; this was suppressed by co-treatment with OTA (Figure 1D). Although treatment with OTA alone marginally enhanced p53 protein generation, AFB_1_-induced p53 expression was suppressed by OTA in a dose-dependent manner (Figure 1E), suggesting that the reduction of Mdm2 expression by OTA may be due to decreased induction of p53 protein production. Additionally, we also assessed aflatoxin-DNA adduct formation, a signature of aflatoxin-induced molecular imprinting, based on the assumption that OTA may antagonize the actions of AFB_1_. AFB_1_ administration increased aflatoxin-DNA adduct formation that was attenuated by co-treatment with OTA (Figure 1F). Our finding demonstrated that OTA antagonized the binding of AFB_1_ to target DNA molecules. Taken together, these results indicated that OTA interfered with molecular events triggered by AFB_1_ including the induction of Mdm2 and p53 expression as well as DNA-adduct formation.

### Antagonistic effects of AFB_1_ and OTA on S phase regulation

**Figure 2:**
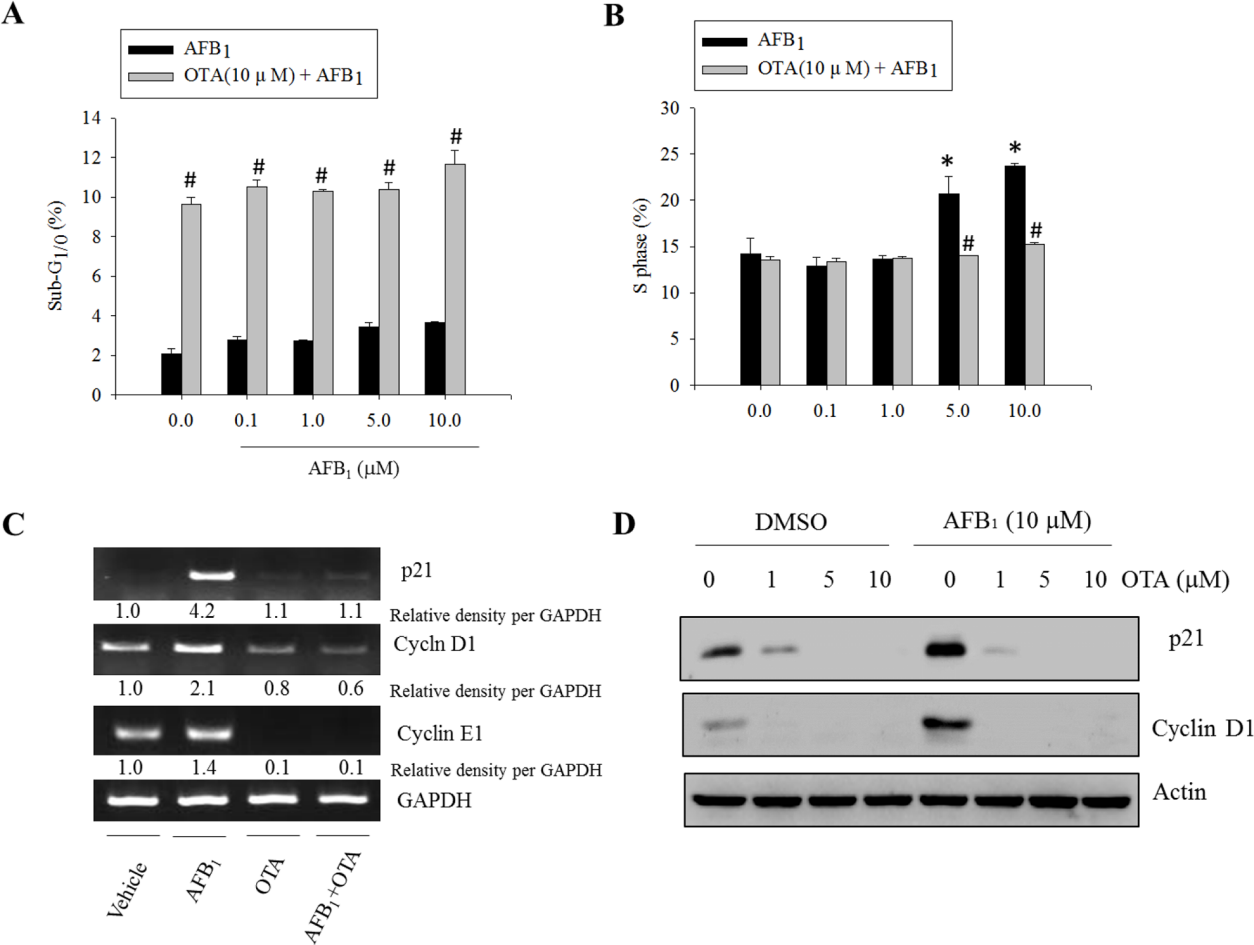
Effects of carcinogenic mycotoxins on the cell cycle in human intestinal epithelial cells HCT-8 cells were treated with different concentrations of AFB_1_ in the presence or absence of OTA (10 μM) for 24 h, and the cells were stained with PI for FACS analysis. **A.** The ratio of cells in the sub-G_1/0_ phase. **B.** The ratio of cells in the S phase. An asterisk (*) indicates a significant difference compared to the control group treated with DMSO alone (*p* < 0.05). A hatch mark (#) indicates a significant difference compared to the group treated with AFB_1_ alone (*p* < 0.05). **C.** HCT-8 cells were treated with AFB_1_ (10 μM), OTA (10 μM), or a combination of the two reagents for 24 h. mRNA expression of each gene was measured using real-time PCR. **D.** HCT-8 cells were treated with various concentrations of OTA in the presence or absence of AFB_1_. Total cell lysates were subjected to Western blot analysis.

As representative functions affected by p53 and Mdm2, the cell death or cell cycle were measured in cells treated with the genotoxic mycotoxins such as AFB_1_ and OTA. Although AFB_1_ (0 − 10 μM) had marginal effects on the induction of apoptosis, a high dose (10 μM) of OTA increased the ratio of cells in the sub-G_0/1_ phase (Figure 2A). In terms of cell cycle, the arrest in the S phase was significantly promoted by AFB_1_ treatment. This effect was attenuated by the presence of OTA (Figure 2B). Moreover, AFB_1_ induced the expression of cell cycle mediators including cyclin-dependent kinase-inhibitor p21^WAF1/CIP1^ (p21), cyclin D1, and cyclin E1. Generation of these factors was almost completely decreased by co-treatment with OTA (Figure 2C and 2D).

**Figure 3:**
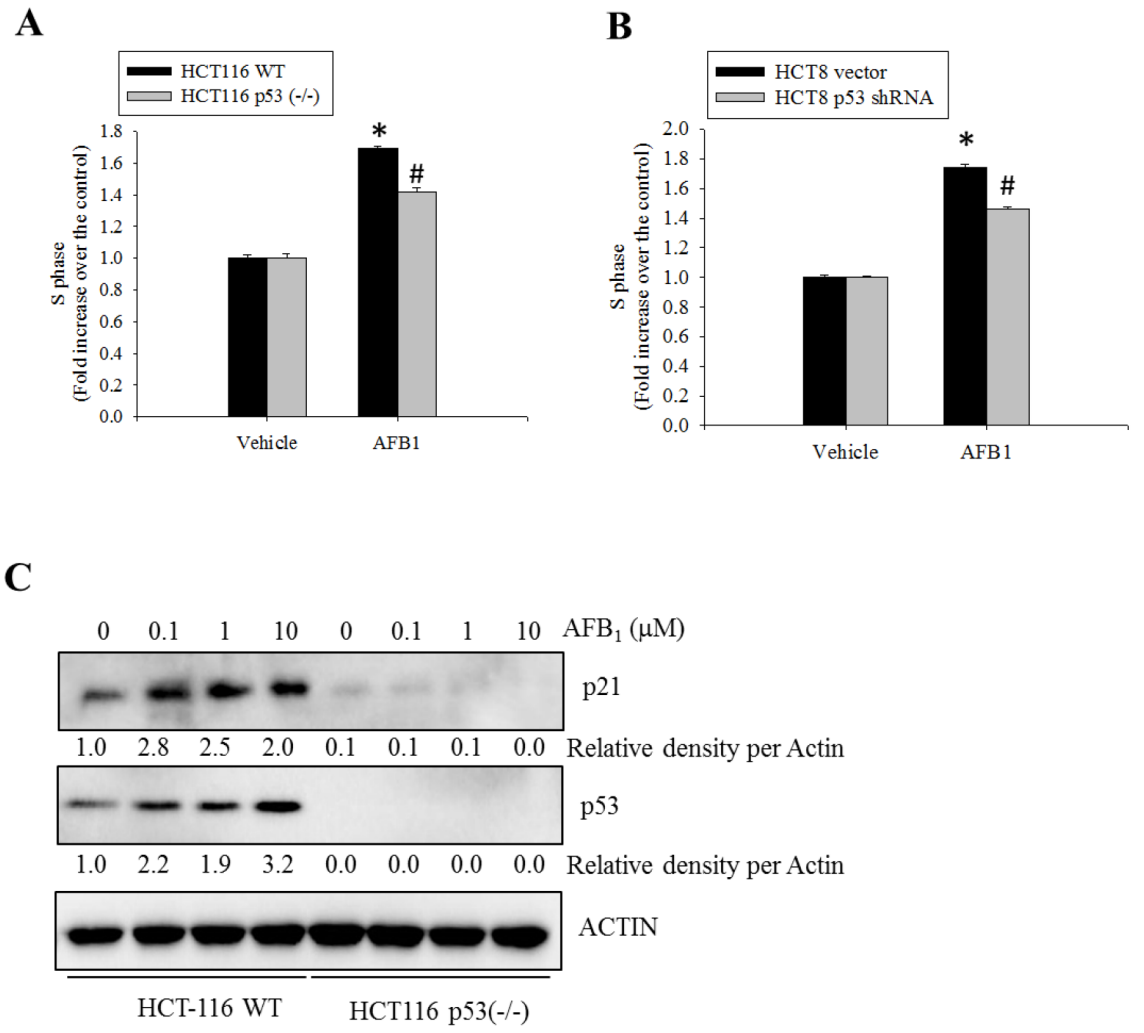
Roles of p53 protein in AFB1-induced S phase arrest **A.** Wild-type or p53^−/−^ HCT-116 cells were treated with DMSO or AFB_1_ (10 μM) for 24 h. The cells were then stained with PI for FACS analysis. **B.** HCT-8 cells transfected with an empty vector or one encoding p53-specific shRNA were treated with AFB_1_ (10 μM) for 24 h, and stained with PI for FACS analysis. An asterisk (*) indicates a significant difference compared to the control wild-type HCT-116 cells (*p* < 0.05). A hatch mark (#) indicates a significant difference relative to wild-type HCT-116 cells treated with AFB_1_ (*p* < 0.05). **C.** Wild-type or p53^−/−^ HCT-116 cells were treated with different concentrations of AFB_1_ for 24 h. Total cell lysates were subjected to Western blot analysis.

To determine whether AFB_1_-induced S phase arrest was mediated by p53, the effects of suppressed p53 expression on cell cycle progression was assessed. Genetic ablation of p53 partially decreased AFB_1_-induced S phase arrest (Figure 3A). Similar effects were observed in cells in which p53 expression was abolished with shRNA (Figure 3B). Although p53 deficiency completely suppressed p53-promoted p21 protein, a well-known mediator of cell cycle arrest (Figure 3C), there is also a possibility that treatment with AFB_1_ led to p53-independent S phase arrest. Taken together, these findings indicate that AFB_1_ induced S phase arrest partly due to increased p53 expression that was prevented by co-treatment with OTA.

### Depletion of CYP3A5 increased AFB_1_-induced DNA adduct

**Figure 4:**
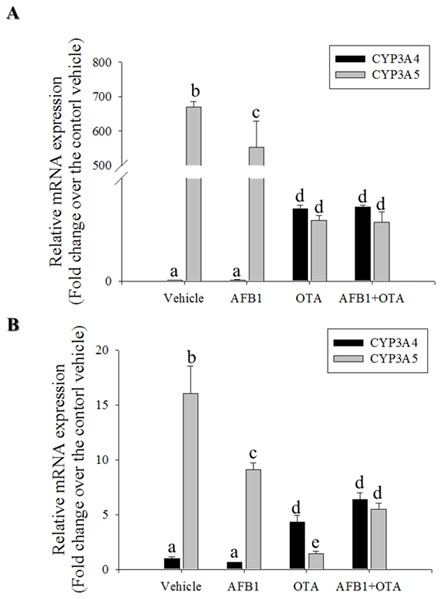
Effects of OTA and AFB1 on intestinal CYP3A **A.** and **B.** HCT-8 (A) or HT-29 (B) cells were treated with 10 mM AFB_1_, 10 mM OTA or their combination for 24 h. mRNA expression of each gene was measured using real-time PCR. Different letters over each bar represent significant differences between groups (p < 0.05).

**Figure 5:**
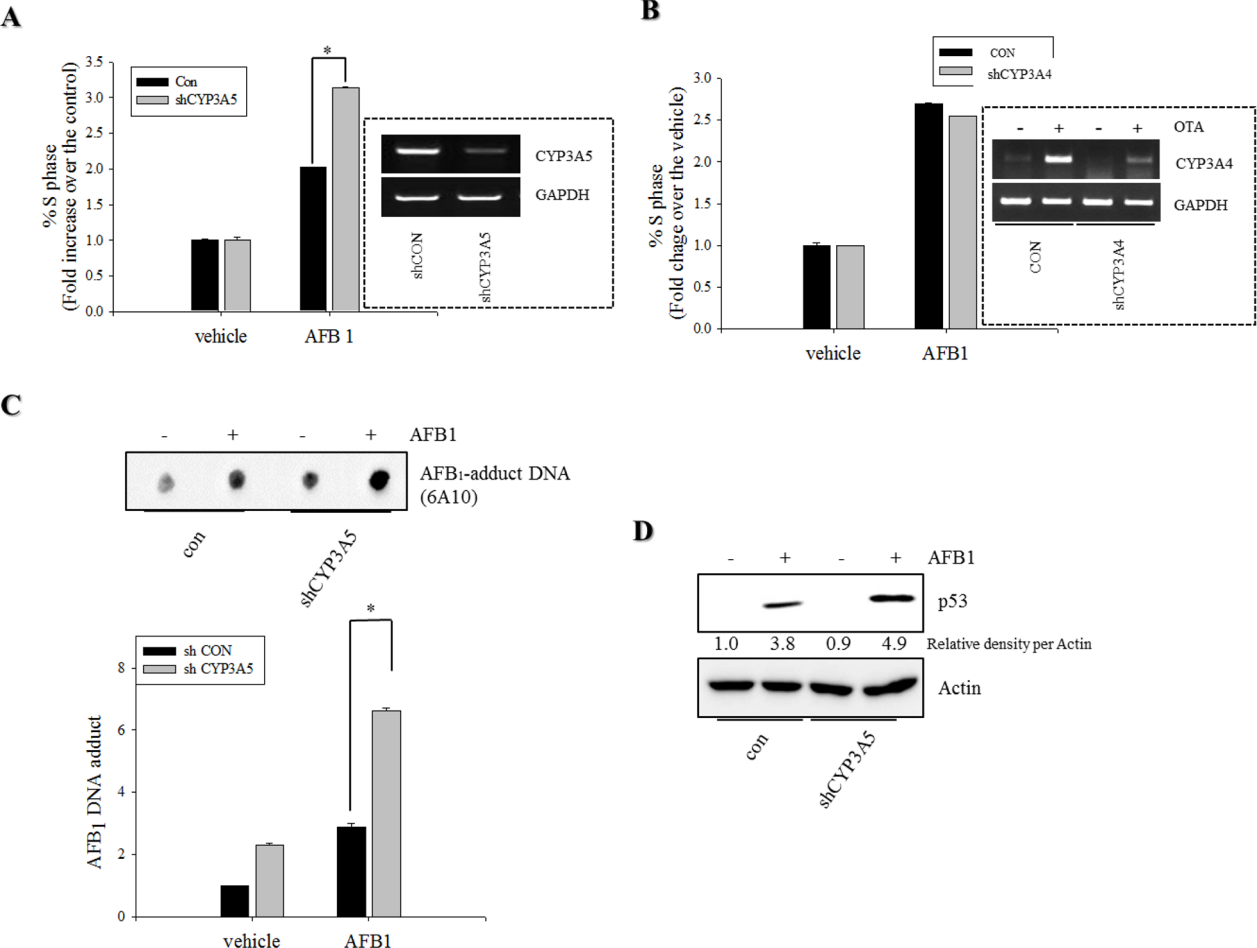
Roles of CYP3A5 gene depletion in S phase arrest and AFB1-DNA adduct in human intestinal epithelial cells **A–B.** HCT-8 cells stably-transfected withempty vector (con) or plasmid for CYP3A4/5 shRNA (shCYP3A5/4) were treated with DMSO or 10 mM AFB_1_ for 24 h. Cells were analyzed for cell cycle according to PI staining followed by FACS analysis. Figures in the box indicate CYP3A5 expression in the stable cell lines. **C.** HCT-8 cells stably-transfected withempty vector or shCYP3A5 were treated with DMSO or 10 mM AFB_1_ for 72 h. AFB_1_ DNA adduct (6A10) were detected using immunodot-blot assay. DNA adduct measured by multi gauge software (bottom panel), respectively. **D.** HCT-8 cells stably-transfected withempty vector or shCYP3A5 were treated with DMSO or 10 mM AFB_1_ for 24 h. Total cell lysates were subjected to Western blot analysis. An asterisk (*) indicates a significant difference compared to AFB_1_-treated, an empty vector-transfected HCT-116 cells (*p* < 0.05).

CYP3A is the most abundant cytochrome P450 subfamily expressed in the small intestine, with an average (or median) specific content from 50 to 70% of spectrally determined total cytochrome P450 content [36, 37]. CYP3A4 and CYP3A5 are the major isoforms expressed in mucosal villus epithelium of the adult small intestine. In the present study, CYP3A mRNA expression was measured in genotoxic mycotoxin-treated HCT-8 cells. Relative amounts of mRNA expression of CYP3A5 was much higher than those of CYP3A4 mRNA in HCT-8 human intestinal cancer cells that originates from ilocecum (Figure 4A). This is consistent with the previous report that intestinal CYP3A4 content gradually decreases from duodenum to jejunum and ileum [37]. HT-29 colorectal adenocarcinoma cells also showed higher expression of CYP3A5 than that of CYP3A4 (Figure 4B). However, OTA treatment increased CYP3A4 mRNA expression, but decreased CYP3A5 mRNA expression in both intestinal cancer cells. By contrast to the impacts of OTA, AFB_1_ led to marginal changes of expression of CYP3A4 whereas it had partially suppressive effects on CYP3A5 mRNA expression in both intestinal cancer cells. Although CYP3A is reported to have a relatively low affinity for AFB_1_ epoxidation, it is primarily involved in AFB_1_ detoxification through formation of less mutagenic AFQ_1_ in mucosa [38]. On an assumption that CYP3A-mediated metabolic inactivation attenuate the genotoxicity of AFB_1_, we assessed the effects of the CYP3A deficiency on AFB_1_-mediated cell cycle arrest. Genetic ablation of CYP3A5 significantly increased the S phase arrest in enterocytes exposed to AFB_1_ (Figure 5A) whereas CYP3A4 had little effects on cell cycle (Figure 5B). Moreover, CYP3A5 deficiency increased the AFB_1_-DNA adduct formation as another readout of genotoxicity, supporting the protective action of CYP3A5 against gut aflatoxicosis (Figure 5C). Consequently, increased genotoxicity by CYP3A5 deficiency led to more cellular arrest in the S phase with elevated p53 levels in the AFB_1_-exposed enterocytes (Figure 5A and 5D). Taken together, all of results indicate that CYP3A5 is mainly detoxification gene on AFB_1_ in human intestinal epithelial cells. In addition, although CYP3A5 expression is reduced by OTA treatment, OTA enhanced CYP3A4 which would account for suppressed AFB_1_-DNA adduct formation in presence of OTA (Figure 1F).

**Figure 6:**
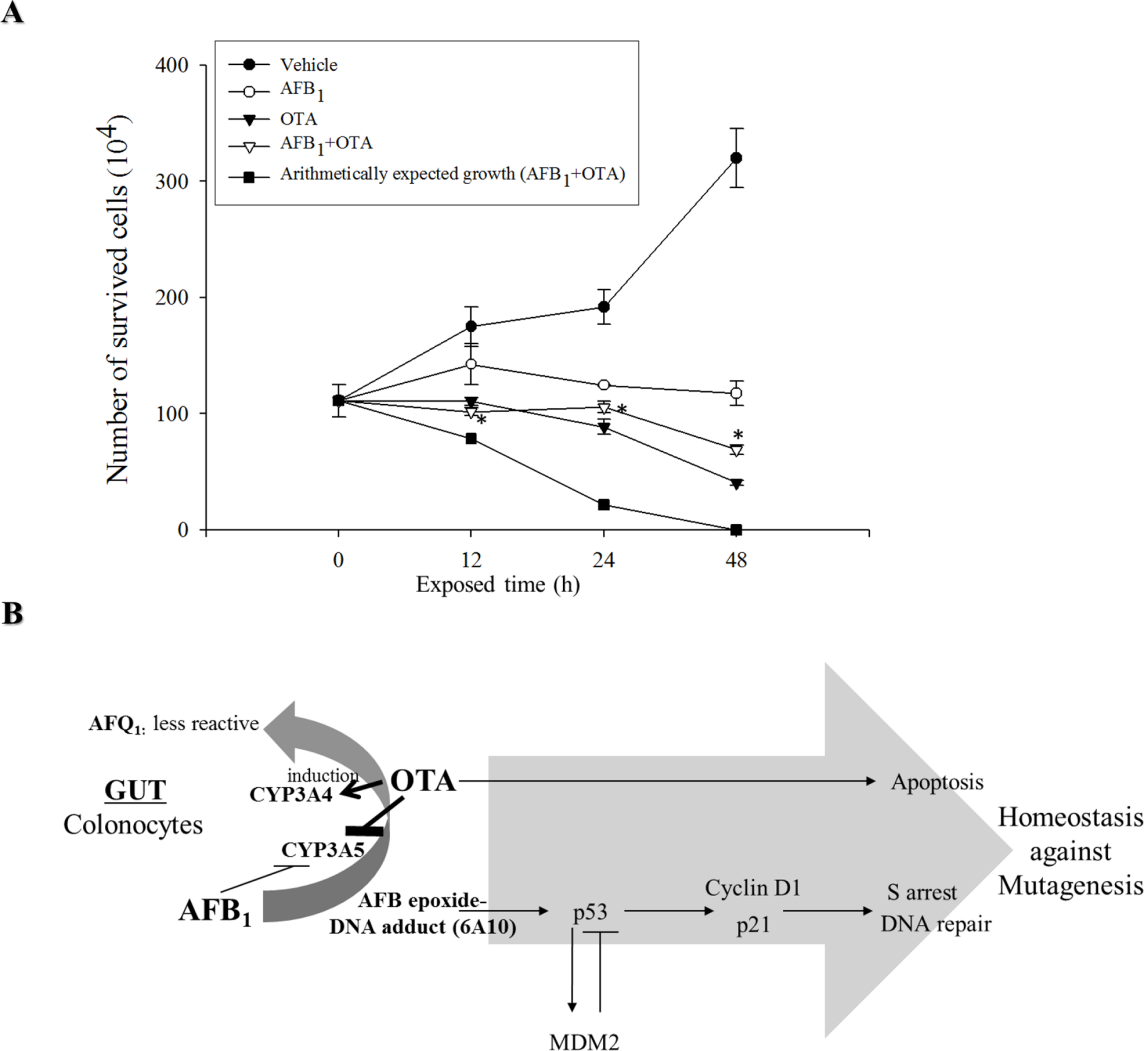
Effects of carcinogenic mycotoxins (AFB1, OTA, or the two in combination) on cell proliferation and putative scheme of growth regulation **A.** HCT-8 cells were treated with AFB_1_ (10 μM), OTA (10 μM), or a combination of the two compounds for different periods of time. Cell survival was quantified and arithmetically expected growth rates were also calculated for cells exposed to AFB_1_ or OTA. An asterisk (*) indicates a significant difference compared to the arithmetically expected values at each time point (*p* < 0.05). **B.** A putative scheme for the antagonistic regulation of cancer cell growth in response to the genotoxic mycotoxin mixture.

Two different regulatory modes including OTA-induced apoptosis and AFB_1_-induced S phase arrest account for decreases in cell proliferation in response to the genotoxic mycotoxins. As expected, single treatment with AFB_1_ or OTA suppressed cellular proliferation (Figure 6A). From the degree of suppression of cell proliferation for the single mycotoxin treatment, the arithmetically-expected levels of proliferation in the presence of both mycotoxins were calculated (Figure 6A). However, the measured levels of experimental proliferation of cells exposed to the mixed mycotoxins were much higher than those expected arithmetic levels, demonstrating the antagonistic interaction between OTA and AFB_1_ on the growth inhibition of intestinal cancer cells.

## DISCUSSION

Cells exposed to carcinogens such as OTA underwent apoptosis which would contribute to the removal of mutated cells in the body. Moreover, treatment with AFB_1_ induced p53 protein expression that was partly associated with S phase arrest which provides times for DNA repair. These growth retardation responses to carcinogenic mycotoxins represent a cellular defense that maintains chromosomal and cellular integrity (Figure 6B). However, OTA treatment antagonized AFB_1_-induced homeostasis response to genotoxic stress. OTA attenuated AFB_1_-triggered cellular arrest, which allow more mutated cells to keep proliferating without falling into cellular arrest required for DNA repair. In detail, co-treatment with these two carcinogenic mycotoxins enhanced epithelial CYP3A4 although epithelial CYP3A5 was decreased by both mycotoxins, resulting in more chance of CYP3A4-mediated metabolic inactivation of AFB_1_ and subsequently reduced formation of AFB_1_-DNA adduct in the cells. As a result, OTA reduced AFB_1_-mediated DNA damages, leading to attenuation of p53-mediated cell cycle checking responses to the mutagens. Therefore, co-treatment with these two carcinogenic mycotoxins poses a greater risk of transformed tumor cell survival due to the disruption of checking responses to mutagens. Mechanistically, this is partly due to decreased levels of tumor suppressor p53, and subsequent enhanced survival and growth of mutated transformed cells without cell cycle retardation (Figure 6B).

Although OTA attenuates AFB_1_-induced cell cycle arrest via suppression of p53, it is also possible that OTA can alter p53-independent cell cycle arrest in AFB_1_-exposed cells. Suppression of tumor formation can be associated with p53-independent cell cycle arrest as well [39, 40]. Although p21, a downstream target of p53-mediated transcription, mediates growth arrest, cellular senescence, and terminal differentiation, p53-independent p21 expression and cell cycle arrest have also been observed [40, 41]. For instance, cell cycle arrest can be mediated by enhanced stabilization of p21 mRNA via protein kinase C [41] or enhanced transcription by release of the p21 promoter from c-Myc-mediated repression [39]. In the present study, AFB_1_-induced p53 production partly contributed to the up-regulation of p21 expression, but it was completely suppressed by OTA treatment. Therefore, p53-independent regulation of p21 expression is also expected to influence cell cycle arrest in response to genotoxic mycotoxins.

p53-dependent responses to DNA damage represent a strong mechanism that protects cells against the accumulation of deleterious mutations [42, 43]. Bypassing p53 activation-linked cellular pathways can therefore make cells susceptible to defective DNA damage and greater genotoxicity. In the present study, AFB_1_-exposed intestinal cancer cells did not undergo G_1_ or G_2_/M arrest despite the expression of wild-type p53. However, S phase arrest occurred partly via a p53-linked pathway. Moreover, co-treatment with OTA and AFB_1_ completely suppressed AFB_1_-induced S phase arrest, strongly implying that more mutated cells can survive genotoxic insults by evading p53-dependent or -independent cell cycle arrest.

A recent study has shown that hepatic cancer cells exposed to AFB_1_ (less than 5 mM) do not develop efficient DNA damage checkpoint responses. This is most likely due to delayed and deficient p53 phosphorylation [44]. Less than 5 mM AFB_1_ does not seem to be sufficient for efficiently inducing a checkpoint response. Cancer cells used in the present study exposed to more than 5 mM AFB_1_ underwent S phase arrest, but these levels of the mycotoxin are far beyond doses (0.015–1 ppm) associated with carcinogenesis in humans and murine models [45, 46]. However, relatively high levels of aflatoxins may exist in the gut luminal environment since the intestinal epithelium is exposed to the entire content of contaminated food [34]. Similar to our study, another investigation also showed that higher doses of AFB_1_ can cause cell cycle arrest [47]. Although AFB_1_ can inhibit the cell cycle and cause S phase arrest, co-treatment with OTA interferes with cell checkpoint regulation and may protect cells against the accumulation of deleterious mutations. Taken together, findings from the present study demonstrate that interference with molecular and cellular checkpoints through the antagonistic activity might allow more mutated cells to withstand co-treatment with two genotoxic mycotoxins, thus increasing the risk of carcinogenesis in humans. Further systematic *in vivo* observations are warranted to more precisely assess the effects of simultaneous exposure to multiple toxins.

Small intestinal CYP has been postulated to be the principal molecule of initial biotransformation of ingested xenobiotics [48]. Among several CYPs expressed in the small intestine, CYP3A is the predominant component, as it is in the liver. Within the small intestine, the duodenum shows the highest expression of CYP3A by northern blot analysis [49]. In previous study, CYP3A4 is reported to have a relatively low affinity for AFB_1_ ep-oxidation, but is primarily involved in AFB_1_ detoxification through AFQ_1_ formation [38]. The present observations show that OTA increased CYP3A4 mRNA expression whereas its basal levels were low, accounting for decreased formation of AFB_1_-DNA adducts. Although intestinal CYP3A4 is robustly induced by OTA treatment, hepatocytes such as HepG2 cells in the present study didn't show OTA-induced CYP3A4 (data not shown), suggesting tissue specific regulation of CYP3A4 by OTA. In spite of CYP3A5 suppressed by OTA or AFB_1_, enhanced CYP3A4 played critical roles in attenuating AFB_1_-induced mutagenesis, leading to less p53 expression. Basal levels of CYP3A5 are much higher than those of CYP3A4 in HCT-8 enterocytes, and thus CYP3A5 contributed to the detoxification of AFB_1_ to some extent in absence of OTA. However, although OTA can decrease the mutagenicity risks of AFB_1_ in enterocytes, OTA itself is another threatening mutagen during gut exposure to the mixture of two genotoxic mycotoxins.

In conclusion, simultaneous exposure to AFB_1_ and OTA lessened the toxic effects of AFB_1_, probably due to metabolic action by CYP3A4. Ultimately, less formation of AFB_1_-DNA adduct led to less p53 expression and subsequently reduced S phase arrest. Although AFB_1_-induced mutation was reduced, the transformed cells had less chance of cell cycle arrest crucial for DNA repair and chromosomal integrity. From the point of view of risk assessment, these findings provide additional information on the co-exposure to two different genotoxic mycotoxins and the mechanism-based evaluation of the mycotoxin interaction. Moreover, the regulatory limits need to be carefully re-assessed, considering the co-exposure and their cross-talking outcome of toxicity.

## MATERIALS AND METHODS

### Cell culture conditions and materials

HCT-116, a human colon cancer cell line and isogenic HCT-116 p53(−/−) cell lines were authenticated and kindly provided by Dr. Bert Vogelstein (Johns Hopkins University, Baltimore, MD, USA) in 2010. HCT-8 and HT29 cells were authenticated by American Type Culture Collection (ATCC) (Manassas, VA, USA) and purchased from ATCC in 2014. The cells were maintained in RPMI medium (Welgene, Daegu, South Korea) supplemented with 10% (v/v) heat-inactivated fetal bovine serum (FBS; Welgene), 50 units/mL penicillin (Welgene), and 50 mg/mL streptomycin (Welgene) in a 5% CO_2_ humidified incubator at 37°C. Cell number and viability were assessed with a standard trypan blue exclusion assay (Sigma-Aldrich, St. Louis, MO, USA). AFB_1_ (≥98% pure according to HPLC, Sigma-Aldrich) was isolated from *Aspergillus flavus*. OTA (≥98% pure) was isolated from *Petromyces albertensis* (Sigma-Aldrich). The p53 shRNA in IMG-803 vector was purchased from IMGENEX (IMGENEX, San Diego, CA, USA).

### Reporter-expressing enterocyte cells

A secretory alkaline phosphatase (SEAP) reporter vector (pSEAP4.14h) was constructed by replacing the *luciferase* gene from the pGL4.14-hygo vector (Promega, Madison, WI, USA) with the *SEAP* gene. The Mdm2 promoter (+580 − +934) in the pGL2-luc vector, kindly provided by Dr. Jill Bargonetti (Hunter College, New York, NY, USA), was cloned into the pSEAP4.14h vector at the *XhoI* and *BglII* sites to produce the pMdm2-SEAP4.14h vector. To create pMdm2-SEAP4.14h-transfected stable cell line, HCT-8 cells were transfected using OmicsFect (Omicsbio, Taipei City, Taiwan) according to the manufacturer's protocol. The stably transfected clones were selected with complete medium containing 400 μg/mL hygromycin B (Invitrogen, Carlsbad, CA, USA). The selected clones were maintained in complete medium supplemented with 200 μg/mL of hygromycin B.

### SEAP assay

Transfected HCT-8 cells were seeded in a 24-well plate at a density 5 × 10^4^ cells/well and then treated with mycotoxins for 24 h at 37°C. The culture medium was collected (400 μL) and heated at 65°C for 5 min. Next, 100 μL of the heated medium was mixed with 100 μL of 2x SEAP assay buffer (2 M diethanolamine, 1 mM MgCl_2_, and 20 mM 1-homoarginine). The mixture was incubated at 37°C for 10 min and the reaction was terminated by adding 20 μL of 120 mM p-nitrophenylphosphate dissolved in 1x SEAP assay buffer. The final mixture was further incubated at 37°C for 15 h in the dark. Absorbance of the reaction mixture was measured at 405 nm with an enzyme-linked immunosorbent assay (ELISA) reader (Molecular Devices, Sunnyvale, CA, USA).

### Western blot analysis

Protein expression levels were compared by a Western blot analysis. Cells were washed with ice-cold phosphate buffer, lysed in boiling lysis buffer (1% [w/v] SDS, 1.0 mM sodium orthovanadate, and 10 mM Tris [pH 7.4]) and sonicated for 5 s. Proteins in the lysates were quantified using a BCA protein assay kit (Pierce, Rockford, IL, USA). Fifty mg of protein were separated by Bio-Rad mini gel electrophoresis (Bio-Rad, Hercules, CA, USA). The proteins were transferred onto PVDF membranes (Pall Corporation, New York, NY, USA). The blots were blocked for 1 h with 5% skimmed milk in Tris-buffered saline plus 0.1% Tween (TBST). Subsequently, the membranes were probed with rabbit polyclonal anti-human actin, mouse monoclonal anti-human p53, or rabbit polyclonal anti-human p21 antibodies (Santa Cruz Biotechnology, Santa Cruz, CA, USA) for 2 h at room temperature or overnight at 4°C. After washing three times with TBST, the blots were incubated with horseradish peroxidase-conjugated secondary antibody for 1 h and washed with TBST three times. Antibody binding was detected with a pico enhanced peroxidase kit (ELPIS Biotech, Daejon, South Korea).

### Reverse transcription (RT) and real-time PCR

RNA was extracted using RiboEX (GeneAll Biotechnology, Seoul, South Korea) according to the manufacturer's instructions. RNA (100 ng) from each sample was transcribed into cDNA using Prime Moloney murine leukemia virus reverse transcriptase (Genetbio, Nonsan, South Korea). cDNA amplification was performed using N-Taq DNA polymerase (Enzynomics, Seoul, Korea) in a MyCycler thermal cycler (Bio-Rad) using the following parameters: initial denaturation at 95°C for 2 min, and varying numbers of cycles of denaturation at 95°C for 30 s, annealing at 58°C for 30 s, and elongation at 72°C for 30 s. An aliquot of each PCR product was subjected to 1% (w/v) agarose gel electrophoresis and visualized by ethidium bromide (EtBr) staining. Sequences of each forward and reverse complement PCR primer were 5′- TCA ACG GAT TTG GTCGTA TT-3′ and 5′- CTGTGG TCA TGA GTC CTT CC-3′ for human GAPDH; 5′- GAG CAG GCA AAT GTG CAA TA-3′ and 5′- GTC CGA TGA TTC CTG CTG AT-3′ for human Mdm2; and 5′- CCC TGG GTG TCC TA TTC AA-3′ and 5′- TGG CAT TTT GA GAG GAA GT-3′ for human cyclin D1.

For real-time PCR, FAM was used as the fluorescent reporter dye and conjugated to the 5′ ends of the probes to detect amplified cDNA with an iCycler thermal cycler (Bio-Rad) using the following parameters: initial denaturation at 94°C for 2 min, and 40 cycles of denaturation at 98°C for 10 s, annealing at 59°C for 30 s, and elongation at 72°C for 45 s. Each sample was tested in triplicate. Relative quantification of gene expression was performed using the comparative threshold cycle (CT) method. The CT value is defined as the point at which a statistically significant increase in fluorescence has occurred. The number of PCR cycles required for FAM intensity to exceed a threshold level just above background was calculated for the test and reference reactions. In all experiments, GAPDH was used as the endogenous control.

### Fluorescence-activated cell sorting (FACS) analysis

Trypsinized cells (1 × 10^6^) suspended in 0.2 mL of PBS and 0.2 mL heat-inactivated FBS were fixed by slowly adding 1.2 mL of ice-cold 70% (v/v) ethanol drop-wise with gentle mixing, and then incubating overnight at 4°C. The cells were washed and incubated in 1 mL propidium iodide (PI) DNA staining reagent (PBS containing 50 μg/mL PI, 50 μg/mL RNase A, 0.1 mM EDTA, and 0.1% [v/v] Triton X-100) on ice until analyzed. The cell cycle distribution was measured with a FACS Calibur apparatus (Becton Dickinson, San Jose, CA, USA). Data for 10,000 cells were collected in the list mode. The 488^th^ line of an argon laser was used to excite the PI, and fluorescence was detected at 615 – 645 nm. The cell cycle of individual cells was studied using a doublet discrimination gating method that eliminates doublets and cell aggregates based on DNA fluorescence. The gate was calibrated to include hypofluorescent cells. Cells in the DNA histogram with hypofluorescent DNA were designated as apoptotic. All other cells had a normal cell cycle profile.

### Genomic DNA isolation

Genomic DNA was extracted using TRIzol reagent (Life Technologies, Carlsbad, CA, USA) according to the manufacturer's instructions. Briefly, the harvested cells were suspended in 1.0 mL of TRIzol reagent, quickly vortexed, and incubated at room temperature for 5 min. The mixture was added to 200 mL of chloroform, shaken vigorously by hand for 15 s, and incubated at room temperature for 3 min. The samples were centrifuged at 13,680 ×g for 15 min at 4°C, and the upper aqueous supernatant was discarded. The samples were then combined with 300 mL of 100% ethanol, mixed by inversion, and incubated for 3 min at room temperature. Next, the samples were centrifuged at 1,870 ×g for 5 min at 4°C, the phenol-ethanol supernatant was discarded, and pellets were washed twice with 1.0 mL of 0.1 M sodium citrate in 10% ethanol by incubating for 30 min at room temperature followed by centrifugation at 1,870×g for 5 min at 4°C. All samples were then re-suspended in 1.5 mL of 75% ethanol and incubated at room temperature for 20 min with intermittent mixing followed by centrifugation at 1,870×g for 5 min at 4°C. The pellets were then air-dried and dissolved in TE buffer (1 mM EDTA and 10 mM Tris-HCl [pH 7.5]). All DNA samples were stored at −80°C until further analysis.

### Immunodot-blot assay for detecting AFB_1_-associated DNA adducts in the genome

To assess the formation of AFB_1_-induced DNA adducts, mycotoxin-treated cells were subjected to an immunodot-blot assay using mouse monoclonal anti-AFB_1_ (6A10) antibody (Novus Biologicals, Inc., Littleton, CO, USA) raised against the open imidazole ring persistent form of the major N7 guanine adduct of AFB_1_. Briefly, heat-denatured DNA was dot-blotted onto a nitrocellulose membrane (Amersham Biosciences, Piscataway, NJ, USA). The membrane was placed over absorbent paper pre-soaked with 0.4 N NaOH for 20 min at room temperature and then blocked overnight at 4°C with 5% skimmed milk in TBST. After multiple washes with TBST, the membrane was incubated with 6A10 antibody (diluted 1:10,000 in 5% skimmed milk with TBST) for 2 h at room temperature. The membrane was washed thoroughly with TBST, incubated with anti-mouse horseradish peroxidase-conjugated immunoglobulin diluted 1:5,000 in 5% skim milk with TBST (Enzo Life Science, Plymouth Meeting, PA, USA) for 2 h at room temperature, and washed with TBST three times. Antibody binding was detected with a pico enhanced peroxidase kit (ELPIS Biotech).

### Statistical analyses

Data were analyzed using SigmaStat for Windows (Jandel Scientific, San Rafael, CA, USA). To compare two groups of data, Student's-*t* test was performed. To compare multiple groups, data were subjected to an ANOVA and pairwise comparisons were made using the Student–Newman–Keuls (SNK) method.

## Abbreviations

IARC: International Agency for Research in Cancer
HCC: hepatocellular carcinoma
AFB_1_: aflatoxin B_1_
AFQ1: aflatoxin Q_1_
OTA: ochratoxin A
MDM2: murine double minute 2 homolog
p53: tumor protein p53
p21: p21^Cip1/Waf1^
CYP3A4: cytochrome P450 3A4
CYP3A5: cytochrome P450 3A5
S phase: synthesis phase

## References

[1] Abrar M, Anjum FM, Butt MS, Pasha I, Randhawa MA, Saeed F, Waqas K (2013). Aflatoxins: biosynthesis, occurrence, toxicity, and remedies. Crit Rev Food Sci Nutr.

[2] Matsuda Y, Wakai T, Kubota M, Osawa M, Sanpei A, Fujimaki S (2013). Mycotoxins are conventional and novel risk biomarkers for hepatocellular carcinoma. World J Gastroenterol.

[3] IARC (1976). Aflatoxins. IARC Monogr Eval Carcinog Risk Chem Man.

[4] Linsell CA (1979). Decision on the control of a dietary carcinogen — aflatoxin. IARC Sci Publ.

[5] McLean M, Dutton MF (1995). Cellular interactions and metabolism of aflatoxin: an update. Pharmacol Ther.

[6] Preston RJ, Williams GM (2005). DNA-reactive carcinogens: mode of action and human cancer hazard. Crit Rev Toxicol.

[7] Aguilar F, Hussain SP, Cerutti P (1993). Aflatoxin B1 induces the transversion of G—>T in codon 249 of the p53 tumor suppressor gene in human hepatocytes. Proc Natl Acad Sci U S A.

[8] Greenblatt MS, Bennett WP, Hollstein M, Harris CC (1994). Mutations in the p53 tumor suppressor gene: clues to cancer etiology and molecular pathogenesis. Cancer Res.

[9] Fuchs R, Radic B, Ceovic S, Sostaric B, Hult K (1991). Human exposure to ochratoxin A. IARC Sci Publ.

[10] Kuiper-Goodman T (1991). Risk assessment of ochratoxin A residues in food. IARC Sci Publ.

[11] Petkova-Bocharova T, Castegnaro M (1991). Ochratoxin A in human blood in relation to Balkan endemic nephropathy and urinary tract tumours in Bulgaria. IARC Sci Publ.

[12] Atroshi F, Biese I, Saloniemi H, Ali-Vehmas T, Saari S, Rizzo A, Veijalainen P (2000). Significance of apoptosis and its relationship to antioxidants after ochratoxin A administration in mice. J Pharm Pharm Sci.

[13] Ranaldi G, Caprini V, Sambuy Y, Perozzi G, Murgia C (2009). Intracellular zinc stores protect the intestinal epithelium from Ochratoxin A toxicity. Toxicol In Vitro.

[14] Assaf H, Azouri H, Pallardy M (2004). Ochratoxin A induces apoptosis in human lymphocytes through down regulation of Bcl-xL. Toxicol Sci.

[15] Pfohl-Leszkowicz A, Manderville RA (2007). Ochratoxin A: An overview on toxicity and carcinogenicity in animals and humans. Mol Nutr Food Res.

[16] Pfohl-Leszkowicz A, Manderville RA (2012). An update on direct genotoxicity as a molecular mechanism of ochratoxin a carcinogenicity. Chem Res Toxicol.

[17] Vogelstein B, Lane D, Levine AJ (2000). Surfing the p53 network. Nature.

[18] Vogelstein B, Kinzler KW (1992). p53 function and dysfunction. Cell.

[19] Otozai S, Ishikawa-Fujiwara T, Oda S, Kamei Y, Ryo H, Sato A, Nomura T, Mitani H, Tsujimura T, Inohara H, Todo T (2014). p53-Dependent suppression of genome instability in germ cells. Mutat Res.

[20] Donehower LA (2014). Insights into Wildtype and Mutant p53 Functions Provided by Genetically Engineered Mice. Hum Mutat.

[21] Vousden KH, Prives C (2009). Blinded by the Light: The Growing Complexity of p53. Cell.

[22] Iwakuma T, Lozano G (2003). MDM2, an introduction. Mol Cancer Res.

[23] Rayburn ER, Ezell SJ, Zhang R (2009). Recent advances in validating MDM2 as a cancer target. Anticancer Agents Med Chem.

[24] Chen X, Qiu J, Yang D, Lu J, Yan C, Zha X, Yin Y (2013). MDM2 Promotes Invasion and Metastasis in Invasive Ductal Breast Carcinoma by Inducing Matrix Metalloproteinase-9. PLoS One.

[25] Noon AP, Vlatkovic N, Polanski R, Maguire M, Shawki H, Parsons K, Boyd MT (2010). p53 and MDM2 in renal cell carcinoma: biomarkers for disease progression and future therapeutic targets?. Cancer.

[26] Mateo EM, Gil-Serna J, Patino B, Jimenez M (2011). Aflatoxins and ochratoxin A in stored barley grain in Spain and impact of PCR-based strategies to assess the occurrence of aflatoxigenic and ochratoxigenic Aspergillus spp. Int J Food Microbiol.

[27] Streit E, Naehrer K, Rodrigues I, Schatzmayr G (2013). Mycotoxin occurrence in feed and feed raw materials worldwide: long-term analysis with special focus on Europe and Asia. J Sci Food Agric.

[28] Moon Y, Yang H, Lee SH (2007). Modulation of early growth response gene 1 and interleukin-8 expression by ribotoxin deoxynivalenol (vomitoxin) via ERK1/2 in human epithelial intestine 407 cells. Biochem Biophys Res Commun.

[29] Moon Y, Yang H, Park SH (2008). Hypo-responsiveness of interleukin-8 production in human embryonic epithelial intestine 407 cells independent of NF-kappaB pathway: new lessons from endotoxin and ribotoxic deoxynivalenol. Toxicol Appl Pharmacol.

[30] Moon Y (2012). Cellular alterations of mucosal integrity by ribotoxins: mechanistic implications of environmentally-linked epithelial inflammatory diseases. Toxicon.

[31] Yang H, Park SH, Choi HJ, Do KH, Kim J, An TJ, Lee SH, Moon Y (2010). Mechanism-based alternative monitoring of endoplasmic reticulum stress by 8-keto-trichothecene mycotoxins using human intestinal epithelial cell line. Toxicol Lett.

[32] Alcantara Warren C, Destura RV, Sevilleja JE, Barroso LF, Carvalho H, Barrett LJ, O'Brien AD, Guerrant RL (2008). Detection of epithelial-cell injury, and quantification of infection, in the HCT-8 organoid model of cryptosporidiosis. J Infect Dis.

[33] Thebault S, Deniel N, Marion R, Charlionet R, Tron F, Cosquer D, Leprince J, Vaudry H, Ducrotte P, Dechelotte P (2006). Proteomic analysis of glutamine-treated human intestinal epithelial HCT-8 cells under basal and inflammatory conditions. Proteomics.

[34] Grenier B, Applegate TJ (2013). Modulation of intestinal functions following mycotoxin ingestion: meta-analysis of published experiments in animals. Toxins (Basel).

[35] Yunus AW, Ghareeb K, Abd-El-Fattah AA, Twaruzek M, Bohm J (2011). Gross intestinal adaptations in relation to broiler performance during chronic aflatoxin exposure. Poult Sci.

[36] Watkins PB, Wrighton SA, Schuetz EG, Molowa DT, Guzelian PS (1987). Identification of glucocorticoid-inducible cytochromes P-450 in the intestinal mucosa of rats and man. J Clin Invest.

[37] Paine MF, Khalighi M, Fisher JM, Shen DD, Kunze KL, Marsh CL, Perkins JD, Thummel KE (1997). Characterization of interintestinal and intraintestinal variations in human CYP3A-dependent metabolism. J Pharmacol Exp Ther.

[38] Eaton DL, Gallagher EP, Bammler TK, Kunze KL (1995). Role of cytochrome P4501A2 in chemical carcinogenesis: implications for human variability in expression and enzyme activity. Pharmacogenetics.

[39] Jeong JH, Kang SS, Park KK, Chang HW, Magae J, Chang YC (2010). p53-independent induction of G1 arrest and p21WAF1/CIP1 expression by ascofuranone, an isoprenoid antibiotic, through downregulation of c-Myc. Mol Cancer Ther.

[40] Zeng YX, el-Deiry WS (1996). Regulation of p21WAF1/CIP1 expression by p53-independent pathways. Oncogene.

[41] Park JW, Jang MA, Lee YH, Passaniti A, Kwon TK (2001). p53-independent elevation of p21 expression by PMA results from PKC-mediated mRNA stabilization. Biochem Biophys Res Commun.

[42] Cipressa F, Cenci G (2013). DNA damage response, checkpoint activation and dysfunctional telomeres: face to face between mammalian cells and Drosophila. Tsitologiia.

[43] Li Q, Zhang Y, El-Naggar AK, Xiong S, Yang P, Jackson JG, Chau G, Lozano G (2014). Therapeutic Efficacy of p53 Restoration in Mdm2-overexpressing Tumors. Mol Cancer Res.

[44] Gursoy-Yuzugullu O, Yuzugullu H, Yilmaz M, Ozturk M (2011). Aflatoxin genotoxicity is associated with a defective DNA damage response bypassing p53 activation. Liver Int.

[45] Besaratinia A, Kim SI, Hainaut P, Pfeifer GP (2009). In vitro recapitulating of TP53 mutagenesis in hepatocellular carcinoma associated with dietary aflatoxin B1 exposure. Gastroenterology.

[46] Liu Y, Wu F (2010). Global burden of aflatoxin-induced hepatocellular carcinoma: a risk assessment. Environ Health Perspect.

[47] Ricordy R, Gensabella G, Cacci E, Augusti-Tocco G (2002). Impairment of cell cycle progression by aflatoxin B1 in human cell lines. Mutagenesis.

[48] Parkinson A, Gemzik B (1991). Production and purification of antibodies against rat liver P450 enzymes. Methods Enzymol.

[49] McKinnon RA, McManus ME (1995). Function and localization of cytochromes P450 involved in the metabolic activation of food-derived heterocyclic amines. Princess Takamatsu Symp.

